# Supplementary material for: MCA: A Multicellular analysis Calcium Imaging toolbox for ImageJ
Source: bioRxiv. 2025 Aug 23:2025.08.19.671108. Preprint. [Version 1] doi: 10.1101/2025.08.19.671108 (PMC12393357; doi:10.1101/2025.08.19.671108)
Supplement: 1 [file NIHPP2025.08.19.671108v1-supplement-1.pdf]

bioRxiv preprint doi: https://doi.org/10.1101/2025.08.19.671108; this version posted August 23, 2025. The copyright holder for this preprint (which was not certified by peer review) is the author/funder, who has granted bioRxiv a license to display the preprint in perpetuity. It is made available under aCC-BY 4.0 International license.

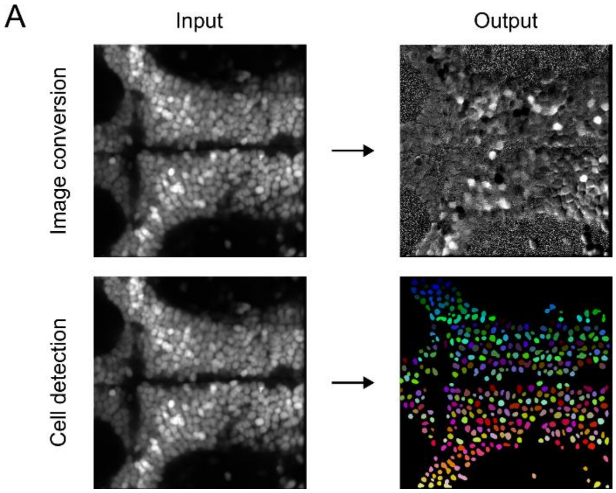

Supplemental figure 1: MCA functions have visual output

A. Representative input image and output image for MCA's image conversion function (top) or Cellpose mediated cell detection (bottom)

bioRxiv preprint doi: https://doi.org/10.1101/2025.08.19.671108; this version posted August 23, 2025. The copyright holder for this preprint (which was not certified by peer review) is the author/funder, who has granted bioRxiv a license to display the preprint in perpetuity. It is made available under aCC-BY 4.0 International license.

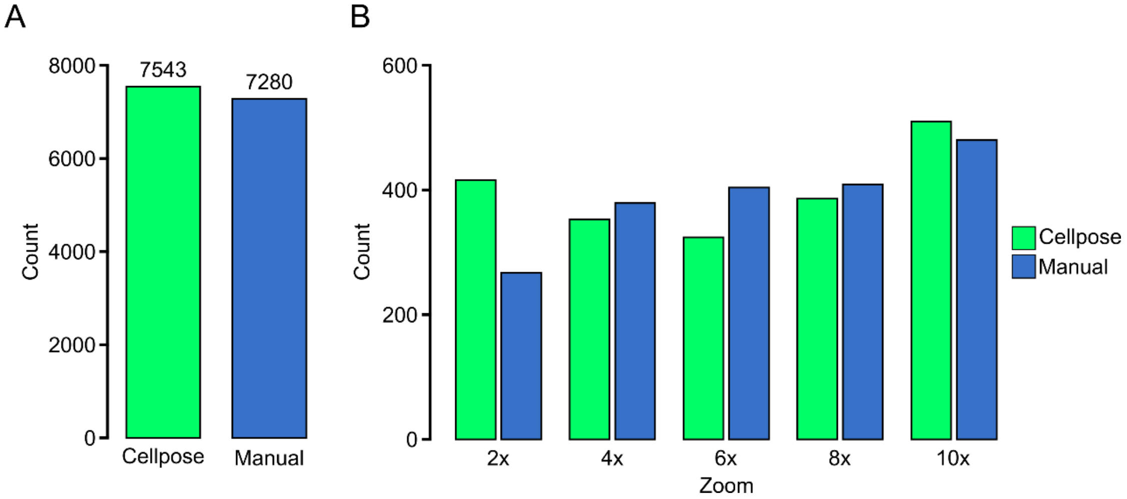

Supplemental Figure 2: Cellpose model verification.

A. Counts of cells in the thalamus extracted from the trained H2B-GCaMP Cellpose model (green, N=19), or manually counted cells (blue, N=19). The number above bar represents the total number of cells counted among all larvae.

B. Comparison of cell counts from Cellpose output, or manual counting based on digital zoom from a 20x water immersion objective.

bioRxiv preprint doi: https://doi.org/10.1101/2025.08.19.671108; this version posted August 23, 2025. The copyright holder for this preprint (which was not certified by peer review) is the author/funder, who has granted bioRxiv a license to display the preprint in perpetuity. It is made available under aCC-BY 4.0 International license.

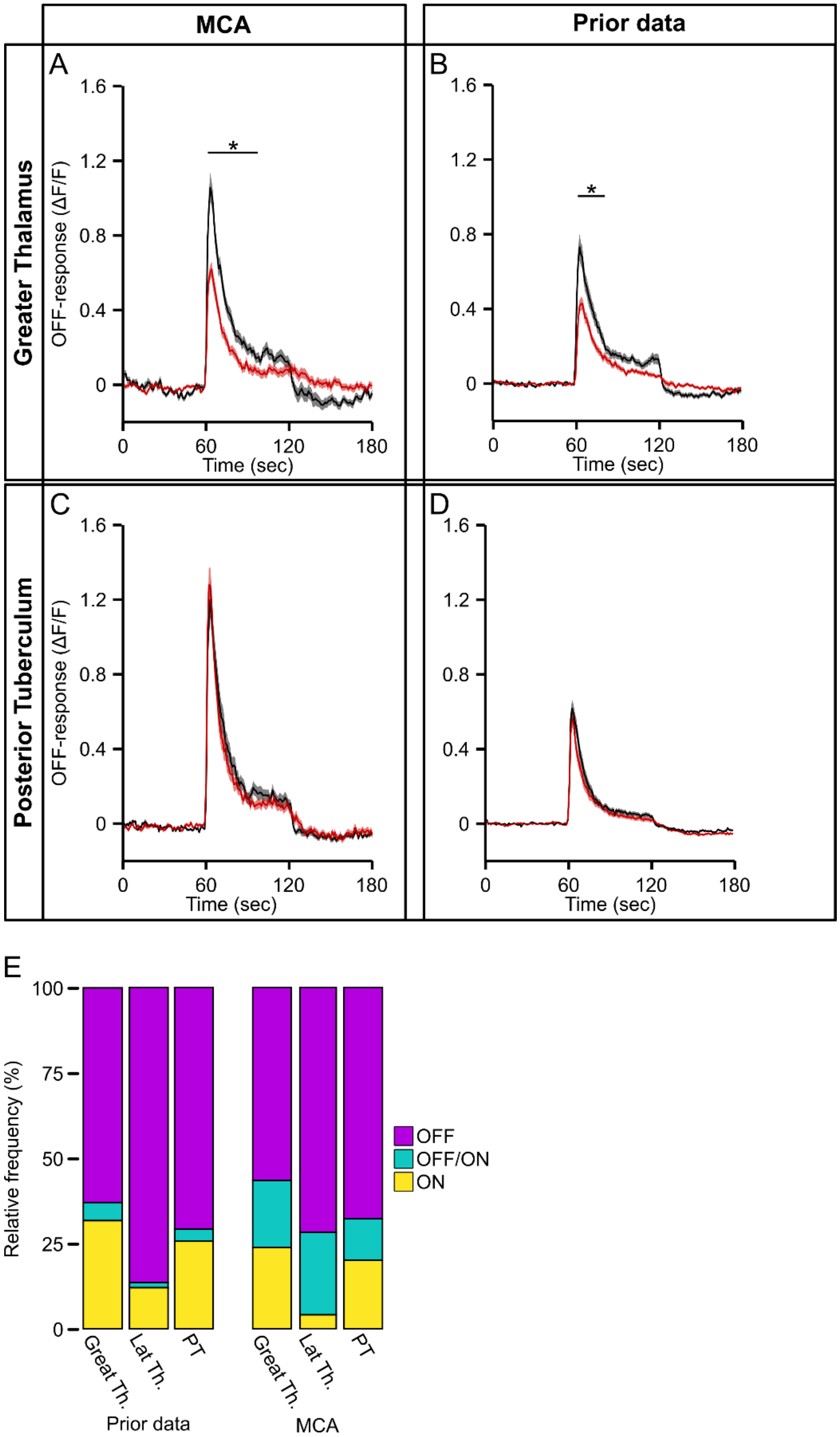

Supplemental Figure 3: MCA data export validation.

A-B. Average response for OFF cells in the Greater Thalamus analyzed with **A.** MCA or **B.** previously published for matched (black) and opposed (red) hemispheres. (MCA: Matched n=84, opposed n=80; Prior data: Matched n=63, opposed n=80).

C-D. Same as in **A-B.** for the Posterior Tuberculum analyzed with **C.** MCA or **D.** previously published (MCA: Matched n=123, opposed n=127; Prior data: Matched n=170, opposed n=148).

bioRxiv preprint doi: https://doi.org/10.1101/2025.08.19.671108; this version posted August 23, 2025. The copyright holder for this preprint (which was not certified by peer review) is the author/funder, who has granted bioRxiv a license to display the preprint in perpetuity. It is made available under aCC-BY 4.0 International license.

E. Relative frequency of cellular response types from each region excluding non-responding cells for OFF (magenta), ON (yellow), or OFF/ON (cyan) responsive cells. * indicates $p<0.05$ two tailed t-test between hemispheres for at least 10 consecutive timepoints.
